# Letters to the President of the Associated Apothecaries, &c. on the Present State of the Practice of Physic and Surgery

**Published:** 1820-09-01

**Authors:** 


*Letters to the President of the Associated Apothecaries and Surgeon-Apothecaries of England and Wales, on the present State of the Practice of Physic and Surgery.* First Series. Anonymous. London, 1820, pp. 77.

The endeavour to increase the respectability of a class of men who are of such essential service to the community at large, as the general practitioners, by a fair statement of their claims to public confidence, merits not only the gratitude of the profession, but of the public in general. Such is the principal object of the very intelligent, although anonymous author of these letters, which are "intended to give a comparative view of particular systems of medical education; to consider the separation of medicine from surgery; to estimate the claims of the general practitioner; and to propose a more respectable mode of remunerating his attendance."

It is very truly stated, that whatever benefit the apothecaries' act has conferred upon the public and the general practitioner, *as a body*, it is inadequate to support individual claim.

"It does not support the interest of the competent practitioner, in the ratio of improvement, in the system of his professional education, which has been enforced by its regulations." "The expensive elementary and professional studies which are required of the candidate for general practice, are highly necessary; and when completed, should secure to him the fair chance of an adequate return."

We most cordially agree with the sentiments of the author contained in the following passage; a radical change in the mode of remunerating the professional attendance of the general practitioner, is most ardently to be desired.

"He *should not* be compelled to seek for payment out of the profits of a trade, wherein he is *obliged* to become a loathsome dealer, as the only method of establishing a charge."

In reference to the fee of the physician, the author observes,

"Whatever difference it may be thought adviseable to make in the *amount* of recompense, for services which are essentially the same, although performed by persons of dissimilar pretensions, the *mode* of remuneration, should unquestionably be founded on a common principle. The general practitioner, equally with the physician, is entitled to be paid for the exercise of his skill."

It should not be forgotten, that a very wide difference exists between the surgeon-apothecary of the present day, and the apothecary fifty years ago. The education of the former is, in most instances, liberal, although graduation at a university, is seldom considered to form a necessary part of it. He enters upon his medical studies, with his mind sufficiently stored with the general principles of knowledge, to reap the well-merited harvest of assiduity; a competent knowledge of the various branches of his profession, by the skilful exertion of which, he afterwards not only reflects credit upon his teachers, but is enabled to confer upon those entrusted to his care, the most valuable benefit that can possibly be derived from human agency;—a diminution of affliction in the trying hour of sickness.

"When the untutored apothecary was merely a pharmacopolist, or at most was *permitted* to prescribe for that numerous part of the community who could not afford to pay the physicians' fee, while he was deemed totally ineligible, to undertake the professional charge of the affluent; when his gains arose exclusively from compounding prescriptions, and neither learning, nor genius, nor skill, were considered necessary qualifications in the medical attendant on the poor, then, indeed, the earnings of his trade were congenial with his pursuits; and in their character, if not always in the sum total of their amount, they would be commensurate with his feelings, and often more than equal to his just desert."

If the public have any compassion upon themselves, they would cheerfully support the proposed alteration in the mode of remuneration, which would effectually prevent the present system of forcing down their throats a quantity of drugs, which are rarely, if ever, necessary. Let it not be forgotten that the proposed reform in the manner of rewarding the services of the general practitioner does *not* contemplate an *increase* of remuneration; on the contrary, to use the words of our author—

"It is probable, that upon a reasonable scheme of recompense, he would receive, in general, a less sum total than heretofore, upon the old trading principle; but would it not be infinitely more gratifying to be paid a fair and moderate compensation in the character of a gentleman and man of science, than in a mode which 'every one concerned for the honour of medicine must reflect on with indignation.'"

That every individual, to whose care the public health is to be confided, should undergo the ordeal of a suitable examination, is very properly insisted upon. The public have a right to require such a proof of the competency of the practitioner, and we sincerely wish we could add, that the examinations which are undergone, are sufficient to satisfy so reasonable a desire. In our opinion, the examinations at the College of Surgeons are much too superficial; they are certainly not commensurate with the highly important duties, the execution of which, the professor of a diploma is authorised to assume. Our author remarks, that "a security against ignorance *is* provided by the examinations which the student is compelled to undergo." Immediately afterwards, however, it is said, that "by these examinations his fitness *should* be accurately determined. The examinations *should* determine, by the strictest enquiry, the knowledge already attained. They should reject incompetency, and silence presumption. They give a passport to merit, and *should* guard the public against imposture."

Now, from the stress laid upon the *should*, we infer that the author is of our opinion; that he is, in fact, very sceptical as to the efficiency of these examinations. In most other countries, the abilities of the candidate for permission to practise are scrutinized with much more severity than in our own;* the consequences which result from such examinations are obvious. The public confidence in the skill of their medical attendant is increased in a ratio proportionate to the strictness of the investigation through which he has passed; while the practitioner himself justly feels proud of those testimonies, which are not bestowed until sufficient proofs are given that they are deserved.

* *Vide*, page 590, in our April Number.

"That a weak and idle pupil does, occasionally, disgrace his school, requires no particular evidence from me; but that he should obtain the certificates of his learned teachers, in testimony either of his diligence or proficiency, demands explanation. That he should be approved by the Court of Examiners, *creates both surprise and regret.*" 38.

The third Letter contains many remarks well worthy the attention of the diplomatized practitioner. We *hope*, that every man who has received into his house a youth, as an apprentice, may peruse the observations of our author upon the duties which devolve upon him, without experiencing any of those "compunctious visitings of nature," which will inevitably intrude upon his mind, if he stand self convicted of not having honourably repaid the confidence reposed in him—of not having endeavoured "to qualify him for the pursuit of those ulterior objects of study, by the full attainment of which he shall be enabled to pass his examinations with credit, and to enter upon the practical duties of his profession with every prospect of benefit to the public." The receipt of a liberal premium, and "a full measure of service from the labour of his apprentice," we *fear*, are sometimes the primary, if not the only object, of the master. If, however, the master performs his duty, we contend, that the period of an apprenticeship is spent in a profitable manner; a thorough knowledge of pharmacy is obtained, without which no man can enter with confidence upon the practical duties of his profession, while the many hours which are unoccupied in the acquirement of pharmaceutical knowledge are, or ought to be, employed in the improvement of those branches of general study, which have previously occupied the attention of the pupil. That he is sometimes permitted to squander away his time without profit to himself, and disgracefully to him to whose charge he is committed, is very certain, and much do we lament the fact. The occasional *non performance* of that which may justly be termed a moral duty on the part of the master, forms no argument, however, in contradiction to the advantages which will be derived from a *well conducted* apprenticeship.

The fourth and last Letter considers the propriety of the division of the healing art into separate departments. The motto chosen by our author is sufficiently expressive of his opinion upon this subject, in which we perfectly concur.

"Omnes medicinæ partes ita connexæ sunt, ut ex toto separari non possint." Celsus.

So long as there exist different grades in the same profession, the duties of the one closely intrenching upon, and, in fact, identifying it with the others, so long will petty jealousies occasionally arise, to the exclusion of those better feelings which alone ought to influence the conduct of the medical practitioner, by whatever name he may be designated. These Letters, however, are not the emanations of professional jealousy; they are unstained by the slightest colouring of illiberality, from which even the liberal professions are not always free. Although the object is to increase the respectability of the general practitioner, no attempt is made to derogate from the claims of the learned and ingenious physician; to him superiority is freely conceded. For our own parts, we disdain the Aristocratic feeling, that plumes itself upon the possession of a title abstractedly considered; and we perfectly coincide in the sentiments of a quotation the author has introduced.

"A physician of a candid and liberal spirit will feel no superiority but what arises from superior learning and superior abilities."— "As a Doctor's degree (says the scientific Gregory)* can never confer sense, the title *alone* can never command respect; neither should the want of it deprive any man of the esteem and deference due to real merit."

The intention of this little pamphlet is deserving of much praise; the unaffected and perspicuous manner in which it is written, evinces the good sense and talent of the author. It will be read with interest by the profession at large, and particularly by the general practitioner, to whose attention it has more immediate claims.

* Dr. Gregory, of Edinburgh.

